# Different Efficacy of Five Soluble Dietary Fibers on Alleviating Loperamide-Induced Constipation in Mice: Influences of Different Structural Features

**DOI:** 10.3390/ijms26031236

**Published:** 2025-01-31

**Authors:** Zhiguo Zhang, Buyu Liu, Wei Liu, Xingquan Liu, Chengcheng Zhang, Weiwei Hu, Weicheng Wu

**Affiliations:** 1 Food Science Institute, Zhejiang Academy of Agricultural Sciences, Hangzhou 310021, China; zhangkii@126.com (Z.Z.); lbyuri@outlook.com (B.L.);; 2 College of Food and Health, Zhejiang Agriculture and Forestry University, Hangzhou 311300, China; 3 Institute of Plant Protection and Microbiology, Zhejiang Academy of Agricultural Sciences, Hangzhou 310021, China

**Keywords:** soluble dietary fiber, structural difference, constipation, defecation function, short-chain fatty acid, gut microbiota

## Abstract

Different dietary fibers have distinct structures, leading to significant variations in their laxative effects. To explore how these structural differences impact constipation intervention, a 14-day study was conducted on loperamide-induced constipated mice using five dietary fibers: soluble dietary fiber from steamed sweet potato (SDF-S), oat β-glucan (OB), polydextrose (PD), arabinogalactan (AG), and inulin (IN). The results showed that four fibers, excluding PD, significantly improved gastrointestinal (GI) transit rate (*p* < 0.05), although PD had the highest fecal moisture, it was significantly different from the lowest IN (*p* < 0.05). AG and IN resulted in higher 6 h fecal weights compared to other fibers. SDF-S and OB were more effective in modulating serum levels of gastrointestinal hormones. The different monosaccharide compositions and glycosidic bonds of these fibers led to distinct changes in gut microbiota composition and SCFA profiles. Galactose and arabinose in AG were linked to increased abundance of *Lachnospiraceae_UCG-006*, *Bacteroides*, and *Odoribacter*, promoting butyrate fermentation, which is positively correlated with GI transit rate. Glucose in SDF-S, OB, and PD favored acetate fermentation positively correlated with fecal moisture. Fructose in IN encouraged the proliferation of *Muribaculaceae_unclassified* and *Ruminococcus*, associated with butyrate fermentation and increased 6 h stool weight, respectively. The β-glycosidic bonds in OB may lead to high butyrate production through the selective proliferation of *Lachnospiraceae_unclassified*. Minor components like fucose, rhamnose, and ribose were positively correlated with the abundance of *Oscillospiraceae_unclassified*, *Anaerotignum*, and *Lachnospiraceae_unclassified*. In conclusion, the unique monosaccharide compositions and glycosidic bond differences in dietary fibers selectively promote the proliferation of fiber-degrading and butyrate-producing bacteria, resulting in varied effects on constipation relief.

## 1. Introduction

Constipation is a highly prevalent functional gastrointestinal (GI) disorder with diverse etiologies. It affects over 10% of the global population, with a higher incidence observed in women and older adults [1,2]. This condition is characterized by persistent difficulties in defecation, incomplete stool evacuation, and hardened stool consistency, which can hinder physical activity, cause discomfort, and lead to psychological distress. These issues collectively diminish overall health-related quality of life. Moreover, chronic or frequent constipation has been associated with an increased risk of serious conditions such as colorectal cancer, Parkinson’s disease, and cardiovascular events, including ischemic stroke and coronary heart disease [3,4]. With the global population aging rapidly, the prevalence of constipation has become an increasingly pressing concern, highlighting the critical need for effective intervention strategies.

Dietary fibers have been used for the treatment of various GI disorders, including constipation, for a long history [5,6]. Dietary fibers can escape digestion in the GI tract and travel to the colon, where they play the role of the bulking agent, helping to maintain high stool water content and resulting in bulky/soft/easy-to-pass stools [7,8]. Furthermore, dietary fibers undergo fermentation by gut microflora, producing a range of metabolites, primarily short-chain fatty acids (SCFA), which can benefit the colon in several ways, including fueling colonic epithelial cells, promoting intestinal peristalsis, lowering intestinal pH, anti-inflammation, and favoring the proliferation of beneficial bacteria [9]. McRoice et al. summarized the physicochemical characteristics and mechanisms underlying the laxative effects of dietary fiber as follows: insoluble fibers with large or coarse particles (e.g., wheat bran) mechanically stimulate the gut mucosa, enhancing the secretion of water and mucus, while gel-forming soluble fibers with high water-holding capacity (e.g., psyllium) prevent dehydration [8]. Additionally, McRoice et al. emphasized that both mechanisms rely on the fiber’s resistance to fermentation and its ability to remain relatively intact throughout the large intestine, noting that soluble fermentable fibers (such as inulin (IN) and fructooligosaccharides) do not possess significant laxative effects.

In recent years, research has increasingly highlighted the anti-constipation activities of soluble dietary fibers derived from various sources with different physicochemical properties, such as inulin [10], *O*-acetylated xylan from bamboo shavings [11], raffino-oligosaccharide [12], chitosan oligosaccharides [13], konjac mannan oligosaccharides [14], among others. These studies have identified dietary fibers’ fermentative metabolism and the resulting changes in gut flora and metabolite profiles as the key mechanism in alleviating constipation. On the other hand, several researchers have suggested that an imbalanced gut flora community is a contributing factor to constipation [15,16]. Furthermore, a bidirectional Mendelian randomization study has provided evidence of a causal relationship between gut microbiota dysbiosis and constipation, particularly involving the carbohydrate metabolizing and butyric acid-producing species [17,18]. These findings strongly support gut microbiota modulation as an effective therapeutic intervention for constipation.

Dietary fibers are a class of carbohydrates with great structural heterogeneity, including the differences in molecular weight, monosaccharide composition, glycosidic linkage, and the consequent variations in solubility, hydration, and solution viscosity. And, different dietary fibers also exhibit diverse gut fermentative properties due to the structure-specific selection of microbial enzymes for the carbohydrate substrate, leading to varying efficacies in mitigating constipation. Lai et al. reported the differing efficacies of four dietary fiber treatments (psyllium husk, polydextrose (PD), wheat bran, and a combination of probiotics) in alleviating constipation symptoms in patients with functional constipation, noting that these interventions were associated with differences in gut microbiota composition [19]. Lu et al. found comparable results in a study involving three dietary fibers (lignocellulose, resistant starch, and konjac flour) in constipated pregnant sows, attributing the varying efficacies to the differences in SCFA profiles resulting from the gut fermentation of these fibers [20]. However, it remains unclear how the structural differences of dietary fibers lead to varied effectiveness in alleviating constipation by mediating different gut microbiota fermentation.

Previously, our team demonstrated that the soluble polysaccharide with better gut fermentative properties was more effective in alleviating loperamide-induced constipation in mice compared to that with lower fermentative capacities [21]. To further investigate the impact of structural differences in dietary fibers on gut microbiota modulation and the improvement of constipation symptoms, five different soluble fibers were selected for in this study: (a) Oat beta-glucan (OB), mainly composed of β-D-glucose polymerized through β-1,3 and β-1,4 glycosidic bonds [22]; (b) PD, a polymer mainly consists of D-glucose linked by α-1,6 glycosidic bonds [23]; (c) IN, predominantly composed of D-fructose linked through β-1,2-fructosyl linkages [24]; (d) Arabinogalactan (AG), a polysaccharide composed of arabinose and galactose, linked by β-1,3 and β-1,6 bonds [25]; (e) Soluble dietary fiber from steamed sweet potato (SDF-S), a resistant dextrin-like fiber with some pectic components, prepared according to our previous study [26]. These dietary fibers were administered to loperamide-induced constipated mice over a 14-day period. The parameters such as defecation function and GI transit, gut microbial structure, and fecal SCFA profiles were compared across the treatment groups. The aim of this research is to provide deeper insights into the specific structures of dietary fibers that influence the development of gut microbiota signatures, which contribute to the recovery from constipation. The findings are expected to improve the efficacy of dietary fiber-based therapies for constipation management.

## 2. Results

### 2.1. Structural Information of the Fibers

The monosaccharide contents and average molecular weights of SDF-S, OB, PD, IN, and AG used in this experiment are exhibited in Table 1. The OB, PD, and SDF-S were mainly composed of glucose, with the contents of 836.51 mg/g, 866.04 mg/g, and 918.48 mg/g, respectively. These dietary fibers also contained various minor sugar components in different amounts, such as mannose, glucuronic acid, galacturonic acid, xylose, arabinose, etc. The IN mostly consisted of fructose (675.83 mg/g) and glucose (278.73 mg/g), together accounting for over 95% of its composition. The AG mainly contained galactose (864.58 mg/g) and a smaller amount of arabinose (99.20 mg/g), with a molar ratio of 7.26:1. The monosaccharide composition aligned with the typical characteristics of these five dietary fibers [22,23,24,25,26]. The OB and AG had relatively higher average molecular weights (101.24 kDa and 48.07 kDa, respectively), while the other three had much lower molecular weights, ranging in 3.01–5.30 kDa. An infrared spectrogram of these five dietary fibers is provided in Supplement Figure S1.

### 2.2. Defecation Function

The defecation function is an important indicator for evaluating the constipation status. In this study, mice were given loperamide to induce constipation for two weeks, simultaneously being administered with one of five soluble fibers or phenolphthalein. The following three defecation and fecal indices, including the total weight of feces in 6 h, stool moisture, and GI transit rate, were assessed to reflect the alleviative effects of different interventions on constipation. As shown in Table 2, the three indices in the MC group were significantly lower than those in the NC group (*p* < 0.05), indicating the decreased defecation function after 14 days of loperamide administration. The five fiber groups (SDF-S, OB, PD, AG, and IN) and the positive treatment group (PT) exhibited significantly increased 6 h stool weights and stool moisture than the MC group (*p* < 0.05). Except for the PD group, the other four fiber groups and the PT group also demonstrated significantly higher GI transit rates than the MC group (*p* < 0.05). These results reflected the alleviative effects of these interventions on typical symptoms of constipation.

These five fibers exhibited varying effects on the defecation parameters. As shown in Table 2, The PD group exhibited significantly higher stool moisture content than the IN group (*p* < 0.05). The fecal moisture levels in the other three dietary fiber groups (SDF-S, OB, and AG) fell between those of the PD and IN groups, while no significant difference was further detected among them. The GI transit rates in the AG, SDF-S, and OB groups were statistically comparable but significantly higher than in the IN group (*p* < 0.05). Conversely, the PD group demonstrated a significantly lower GI transit rate compared to the other four fiber groups (*p* < 0.05). Although no statistical difference was observed in the 6 h stool weight among the fiber groups, the AG and IN groups showed a significantly increased 6 h stool weight compared to the control group (*p* < 0.05). In contrast, the other three fiber groups did not exhibit such an effect, suggesting a superior impact of AG and IN on stool excretion. These findings imply that the fibers influence the defecation function of constipated mice through different mechanisms and to varying degrees.

### 2.3. Gastrointestinal Hormone Levels

GI motility regulatory hormones were investigated for further evaluating the differences between these fibers on intestinal propulsion. In this study, three excitatory (motilin (MTL), gastrin (GAS), and substance P (SP)) and two inhibitory hormones (vasoactive intestinal peptide (VIP), and somatostatin (SS)) in serum were determined and reported in Table 3. Compared with the NC group, a significant increase in the SP level and a significant decrease in the VIP and SS levels were observed in the MC group (*p* < 0.05). A declining tendency in GAS level in the MC group was also observed.

The changes in serum levels of GAS, SP, VIP, and SS in constipated mice were reversed by the intervention of PT and the five fibers. As shown in Table 3, significantly higher levels of serum SP and GAS were detected in the PT and most fiber groups compared to the MC group (*p* < 0.05), with the exception of the SP level in the IN group and the GAS levels in the PD and AG groups. Serum VIP and SS levels in the PT and fiber groups were significantly lower than those in the MC group (*p* < 0.05), except for the VIP level in the AG group. Among the five fiber groups, the SDF-S group demonstrated the highest GAS and SP levels and the lowest SS level. The OB group exhibited the lowest VIP level. Meanwhile, the AG group showed the lowest GAS level and the highest VIP level. The IN group presented the lowest SP level and the highest SS level. These results suggest that the fibers differ in their efficacy and mechanisms for promoting GI motility.

### 2.4. Fecal SCFA Profiles

**Figure 1:**
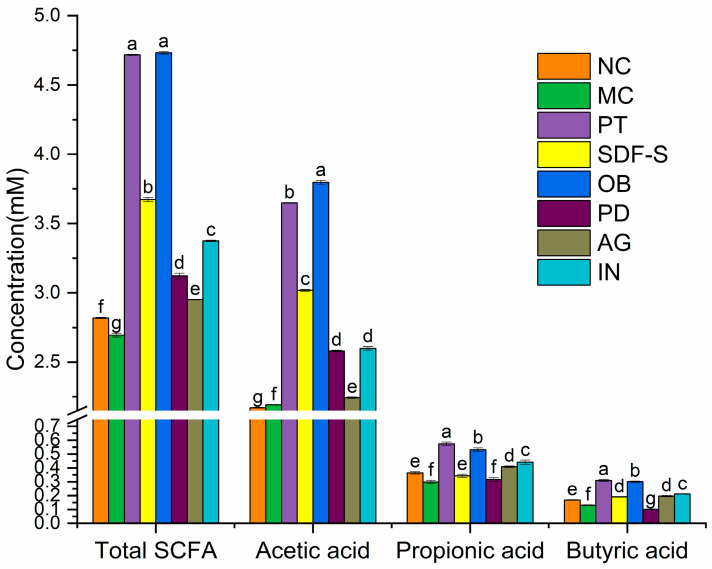
SCFA profiles in mice feces. Different lowercase letters above the error bars indicate significant differences at *p* < 0.05.

The contents of SCFA, including acetic, propionic, and butyric acid were measured in the fresh feces of mice after the 14-day intervention. The fecal SCFA profiles were represented by the total SCFA content and the individual concentrations of acetic, propionic, and butyric acids, as illustrated in Figure 1. The MC group exhibited significantly reduced levels of total SCFAs, as well as propionic and butyric acids, but showed a notably higher fecal concentration of acetic acid compared to the NC group (*p* < 0.05). In contrast, the PT group demonstrated significantly increased total SCFA levels and higher concentrations of all three individual SCFAs compared to the MC group (*p* < 0.05).

Dietary Fiber intervention significantly altered the fecal SCFA profile in constipated mice. As shown in Figure 1, compared to the MC group, the five fiber groups exhibited notably increased total SCFA and acetic acid levels (*p* < 0.05). However, significant enhancement in fecal propionic and butyric acid concentrations was observed only in the fiber groups excluding the PD group (*p* < 0.05). Additionally, the fecal butyric acid level in the PD group was significantly lower than that of the MC group (*p* < 0.05).

Complex variations in the SCFA profile were observed among the five fiber groups, as illustrated in Figure 1. Specifically, the total SCFA and acetic acid levels followed the order: OB > SDF-S > IN > PD > AG, with significant differences among the fiber groups for these two parameters (*p* < 0.05), except for the statistically equivalent acetic acid levels in the PD and IN groups. The fecal propionic and butyric acid concentrations ranked as follows: OB > IN > AG > SDF-S > PD. Significant differences in fecal propionic and butyric acid levels were detected among the groups (*p* < 0.05), except for the butyric acid concentration between the SDF-S and AG groups.

### 2.5. Gut Microbiota Structure

#### 2.5.1. Diversity of Gut Microbial Communities

**Figure 2:**
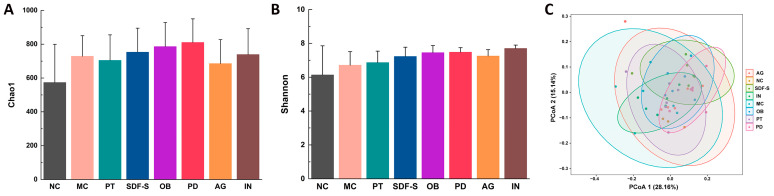
Phylogenetic diversity analysis of gut microbiota in mice. Alpha-diversity analysis indexes include (**A**) Chao1, (**B**) Shannon, and (**C**) beta-diversity analysis indexes including principal coordinate analysis (PCoA) based on the weighted UniFrac metric.

The microbiota diversity of the mice gut was evaluated by 16S rDNA gene sequencing. Community richness was characterized by the Chao1 index (Figure 2A), and community diversity was characterized by the Shannon index (Figure 2B). The differences in the average values of these two indices among groups suggested that fiber intervention had the potential to promote community richness and diversity, though the differences were not statistically significant due to the relatively short intervention duration. The beta diversity was shown by principal coordinate analysis (PCoA)based on the weighted UniFrac metric algorithm (Figure 2C). There was an evident separation among the five fiber groups, indicating that the different types of fiber resulted in diverse gut microbial changes.

#### 2.5.2. Community Structure of Gut Microbiota

**Figure 3:**
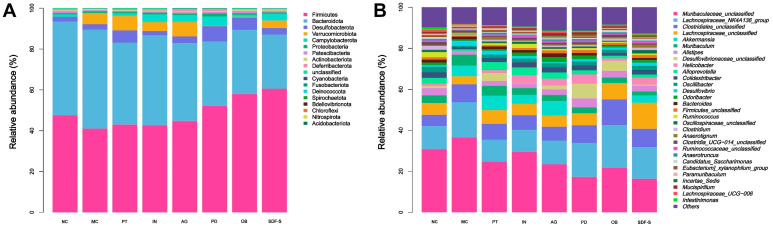
Relative abundance of dominant groups in the gut microbiota of mice at the (**A**) phylum and (**B**) genus levels.

The gut microbial composition was analyzed at the phylum level, as depicted in Figure 3A. The majority of the microbial population consisted of Firmicutes and Bacteroidota, accounting for over 80% of the relative abundance of the microbial community. The results also indicated a decreased abundance of Firmicutes (47.56% vs. 40.98%) and an increased richness of Bacteroidota (45.90% vs. 51.26%) in the MC group compared to the NC group. Dietary fiber interventions elevated the abundance of Firmicutes and decreased that of Bacteroidota. The SDF-S group exhibited the largest increase in Firmicutes and the most substantial decline in Bacteroidota, followed by the OB, PD, AG, and IN groups in turn. Significant differences were observed in the abundance of Bacteroidota between the four fiber groups (SDF-S, OB, PD, and AG) and the MC group (*p* < 0.05). Other bacteria with over 1% relative abundance included Desulfobacterota, Verrucomicrobiota, Campylobacterota, Proteobacteria, Patescibacteria, Deferribacterota, as well as other unclassified phyla.

**Figure 4:**
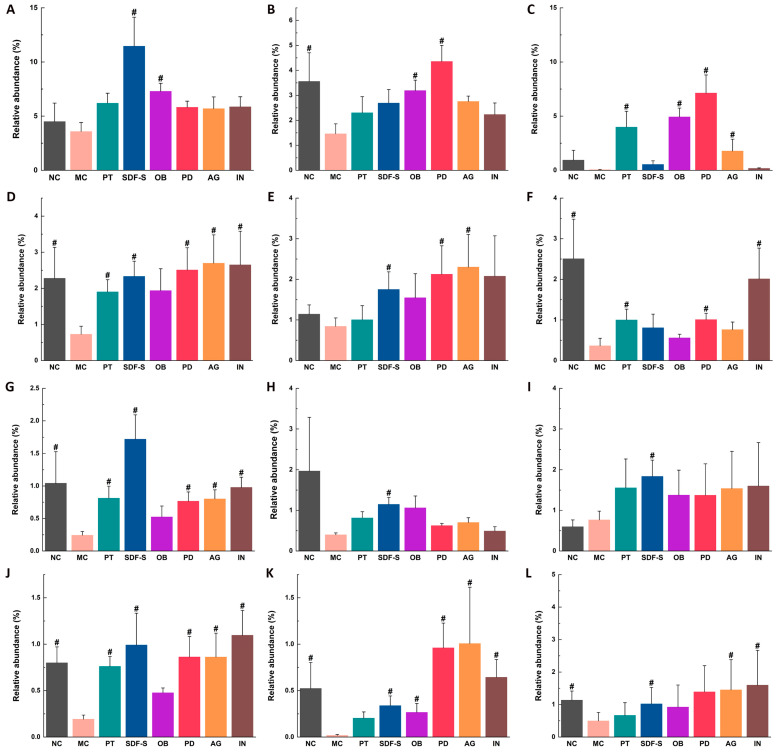
Relative abundance of (**A**) *Lachnospiraceae_unclassified*, (**B**) *Alistipes*, (**C**) *Desulfovibrionaceae_unclassified*, (**D**) *Oscillibacter*, (**E**) *Firmicutes_unclassified*, (**F**) *Ruminococcus*, (**G**) *Oscillospiraceae_unclassified*, (**H**) *Clostridium*, (**I**) *Anaerotignum*, (**J**) *Ruminococcaceae_unclassified*, (**K**) *Mucispirillum*, and (**L**) *Intestinimonas* genera in the gut microbiota of mice. # indicates significant differences between the MC and other groups (*p* < 0.05).

The top 30 genera with the highest relative abundance were selected to analyze the effects of different dietary fiber interventions on gut microbial composition in constipated mice. The richness differences at the genus level are presented in Figure 3B. The results were further analyzed using the Kruskal–Wallis method, and the relative abundance of 12 genera was significantly affected by the experimental interventions, as shown in Figure 4.

Compared to the NC group, the MC group exhibited significant reductions in the relative abundance of seven genera, including *Alistipes*, *Oscillibacter*, *Ruminococcus*, *Oscillospiraceae_unclassified*, *Ruminococcaceae_unclassified*, *Mucispirillum*, and *Intestinimonas* (Figure 4B,D,F,G,J–L) (*p* < 0.05). The effects of dietary fibers on gut flora were evaluated by comparing the microbial composition of the five fiber groups with that of the MC group. The results indicated that all five fibers enhanced the richness of *Mucispirillum* in the constipated mice’s gut (Figure 4K) (*p* < 0.05). Except for the OB, the other four fibers significantly increased the relative abundance of *Oscillibacter*, *Oscillospiraceae_unclassified*, and *Ruminococcaceae_unclassified* (Figure 4D,G,J) (*p* < 0.05). The abundance of *Desulfovibrionaceae_unclassified* in the constipated mice was enhanced by the OB and PD (Figure 4B) (*p* < 0.05), *Oscillospiraceae unclassified* by the PD and IN (Figure 4F) (*p* < 0.05), and *Intestinimonas* by the SDF-S, AG, and IN (Figure 4L) (*p* < 0.05).

Six other genera in the constipated mice’s gut were also significantly increased by different fibers, although the richness of these genera did not differ statistically between the NC and MC groups. Specifically, the OB increased the abundance of *Lachnospiraceae_unclassified* and *Desulfovibrionaceae_unclassified* (Figure 4A,C) (*p* < 0.05). And, the SDF-S enhanced the abundance of *Lachnospiraceae_unclassified*, *Firmicutes_unclassified*, *Clostridium*, *Anaerotignum*, and *Intestinimonas* (Figure 4A,E,H,I,L) (*p* < 0.05). Both PD and AG enriched *Desulfovibrionaceae_unclassified* and *Firmicutes_unclassified* (Figure 4C,E) (*p* < 0.05). Additionally, the AG and IN also increased the relative abundance of *Intestinimonas* (Figure 4L) (*p* < 0.05).

### 2.6. Gut Microbiota Functional Prediction

**Figure 5:**
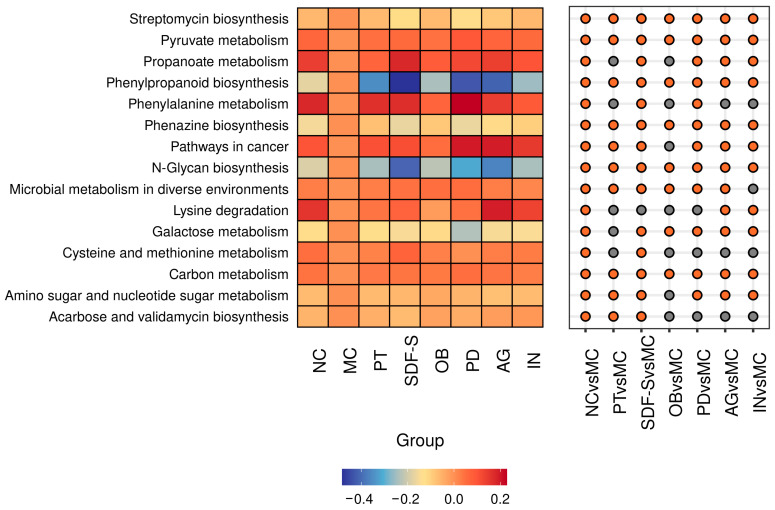
Differences in the mean proportion of the pathways in gut microbiota are shown in the Heatmap Bubble matrix. Heatmap (Left): Predicting based on the logarithmized ratio of each group’s pathway mean proportion compared to the MC group. Bubble Matrix (Right): An orange bubble indicates a significant difference compared to the MC group at *p* < 0.05, while a grey bubble indicates no significant difference.

Phylogenetic Investigation of Communities by Reconstruction of Unobserved States (PICRUSt) analysis was conducted to estimate the impact of the experimental interventions on functional gene-related pathways. According to 355 Keyoto Encyclopedia of Genes and Genomes (KEGG ) pathways, thirteen pathways in the gut microbiota community of mice were significantly altered due to constipation. These pathways are primarily involved in carbohydrate and amino acid metabolism, glycan biosynthesis and metabolism, biosynthesis of other secondary metabolites, and global overview maps. Differences in the mean proportion of these pathways among the groups are illustrated in Figure 5.

As reported in Figure 5, one metabolism pathway (amino sugar and nucleotide sugar metabolism) and five biosynthesis pathways (streptomycin biosynthesis, phenylpropanoid biosynthesis, phenazine biosynthesis, N-glycan biosynthesis, and acarbose and validamycin biosynthesis) were significantly upregulated in the MC group (*p* < 0.05). In contrast, seven other pathways, mainly involving sugar and amino acid metabolism, were significantly downregulated (*p* < 0.05). These changes in the constipated mice were reversed by the intervention with the five dietary fibers used in this study. Notably, SDF-S showed unique effects on the pathways of acarbose and validamycin biosynthesis and amino sugar and nucleotide sugar metabolism (*p* < 0.05). Only AG and IN interventions impacted the lysine degradation pathway (*p* < 0.05), while SDF-S and PD affected the phenylalanine metabolism pathway (*p* < 0.05). Except for the OB, the other four dietary fibers had significant effects on the pathways of propanoate metabolism and amino sugar and nucleotide sugar metabolism (*p* < 0.05).

### 2.7. Correlation Analysis

**Figure 6:**
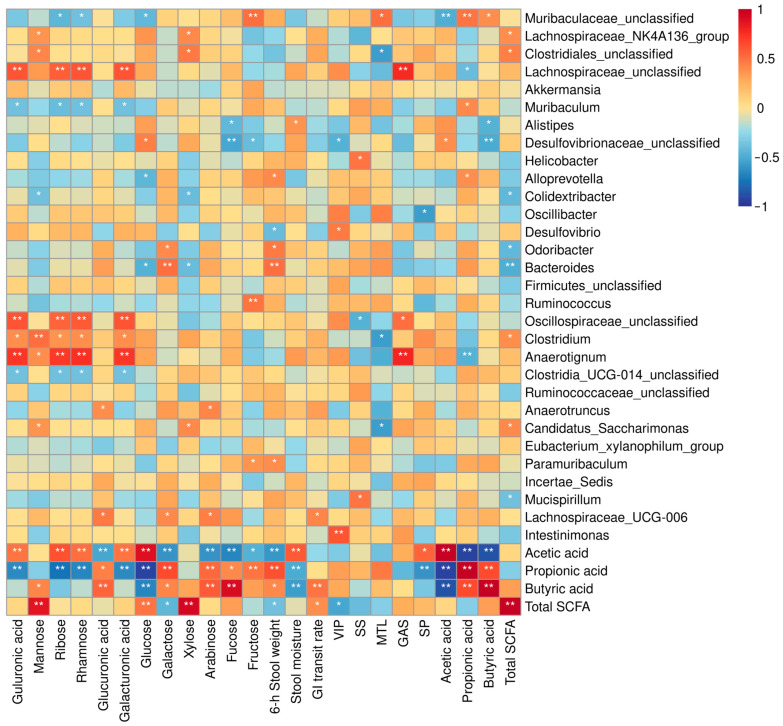
Correlation analysis among monosaccharide concentration, defection parameters, fecal SCFA profile, and relative richness of top 30 genera of gut microbiota. A significance of *p* < 0.05 indicated as *, and *p* < 0.01 indicated as **.

Pearson correlation analysis was performed to explore the relationships among defecation parameters, constipation-related hormones, the top 30 genera of gut flora, and fecal SCFAs. In the analysis, the ratios of acetic, propionic, and butyric acids to total SCFAs were calculated as variables, as previous research has demonstrated that SCFA ratios are more sensitive indicators for evaluating their effects on constipation status [27]. The results are presented as a heatmap in Figure 6.

Defecation parameters exhibited broad correlations with SCFA profiles and gut microbiota. The GI transit rate was positively correlated with the fecal butyric acid ratio and total SCFA levels (*p* < 0.05). Stool moisture was negatively associated with the propionic and butyric acid ratios (*p* < 0.05) but positively correlated with the acetic acid ratio (*p* < 0.05). The 6 h stool weight was positively correlated with the propionic and butyric acid ratios (*p* < 0.05) but negatively associated with the acetic acid ratio and total SCFA content (*p* < 0.05). Additionally, these defecation parameters were positively correlated with the relative abundance of six bacterial genera, including *Lachnospiraceae_UCG-006*, *Paramuribaculum*, *Bacteroides*, *Odoribacter*, *Alloprevotella*, and *Alistipes* (*p* < 0.05). Moreover, constipation-related hormones, comprising three excitatory (MLT, GAS, and SP) and two inhibitory hormones (VIP and SS), exhibited intricate relationships with the gut microbiota, with twelve bacterial genera showing significant correlations (*p* < 0.05), as shown in Figure 6. Furthermore, total SCFA levels were significantly and negatively correlated with serum VIP levels (*p* < 0.05).

Dietary fibers, as substrates for gut microbiota fermentation, have their monosaccharide composition closely linked to gut flora composition and fecal SCFA profiles. In this study, positive correlations were observed between arabinose and *Lachnospiraceae_UCG-006* and *Anaerotruncus* (*p* < 0.05), as well as between galactose and *Lachnospiraceae_UCG-006*, *Odoribacter*, and *Bacteroides* (*p* < 0.05). Glucose showed a positive correlation with *Desulfovibrionaceae_unclassified* (*p* < 0.05) but was negatively associated with *Bacteroides* and *Alloprevotella* (*p* < 0.05). Notably, minor monosaccharides such as guluronic acid, ribose, rhamnose, and galacturonic acid exhibited positive correlations with the abundance of beneficial bacteria, including *Anaerotignum*, *Clostridium*, *Oscillospiraceae_unclassified*, and *Lachnospiraceae_unclassified* (*p* < 0.05). These findings underscore the critical role of these minor components in shaping gut microbiota composition, even though their concentrations are relatively low.

Among the four main monosaccharide components of dietary fiber studied, glucose showed a positive correlation with the fecal acetic acid ratio (*p* < 0.05) but a negative correlation with the propionic and butyric acid ratios (*p* < 0.05). Conversely, arabinose and galactose were positively correlated with the propionic and butyric acid ratios (*p* < 0.05) but negatively correlated with the acetic acid ratio (*p* < 0.05). Fructose also exhibited a negative correlation with the acetic acid ratio (*p* < 0.05) and a positive correlation with the propionic acid ratio (*p* < 0.05). Among the less abundant monosaccharide components, glucuronic acid shared a similar correlation pattern with galactose for the fecal acetic, propionic, and butyric acid ratios (*p* < 0.05). Meanwhile, guluronic acid, ribose, rhamnose, and galacturonic acid showed a positive correlation with the acetic acid ratio (*p* < 0.05) and a negative correlation with the propionic acid ratio (*p* < 0.05). Mannose was positively correlated with the butyric acid ratio (*p* < 0.05). Additionally, glucose, mannose, and xylose were positively correlated with total SCFA production (*p* < 0.05), whereas galactose was negatively correlated with total SCFA production (*p* < 0.05).

## 3. Discussion

Dietary fiber supplementation is the first-line treatment for chronic constipation according to British, American, and European guidelines [28,29,30]. The alleviating effects of dietary fibers on constipation involve several mechanisms, with particular emphasis on their ability to modulate gut microbiota and metabolite composition. This is of great interest, as an imbalanced gut microbiota is now considered one of the etiological factors contributing to chronic constipation. However, different dietary fibers possess distinct physicochemical structures and gut fermentation properties, which, in turn, influence the structure of the gut microbiota and the composition of fermentation products. This has prompted increased interest in exploring the relationship between the structure of dietary fibers and their effectiveness in alleviating constipation. Therefore, we selected five dietary fibers with varying monosaccharide compositions and glycosidic bond structures and then compared their effects on a loperamide-induced mouse model of constipation. We found that the five dietary fibers improved the defecation function in constipated mice with different extents and on varied aspects. Furthermore, we explored the reasons behind these differences from the perspective of gut microbiota fermentation differences. This study highlights that the structural characteristics of dietary fibers are crucial in determining their therapeutic efficacy in constipation intervention. Our findings underscore the importance of considering the specific properties of dietary fibers when designing and recommending treatments for chronic constipation.

Constipation is typically characterized by impaired colonic motility, leading to infrequent defecation and hard stools. In this study, constipated mice exhibited a lowered GI transit rate, decreased 6 h stool weight, and reduced stool moisture. The 14-day dietary fiber treatments increased the GI transit rate in the constipated mice, except for the PD treatment (Table 1). Significant differences were observed among the other four fiber treatments, ranked as follows: AG > OB = SDF-S > IN. However, the PD treatment showed a tendency to increase stool moisture more than the other four dietary fibers. When considering the 6 h fecal weight, AG and IN treatments demonstrated a slight superiority over the other three dietary fibers. The findings suggest that these dietary fibers affect different aspects of defecation function, with varying degrees of effectiveness.

Dietary fiber has been found to increase fecal volume, mechanically stimulating intestinal motility, and shortening colonic transit time [31]. Previous research suggests that viscous, gel-forming soluble polysaccharides with low fermentability and the ability to increase fecal volume are key to effectively promoting bowel movements [8]. Among the five dietary fibers included in this study, only OB and AG are viscous polysaccharides with gel-forming capabilities, while the non-viscous polysaccharides such as SDF-S and IN also demonstrated notable efficacy in promoting defecation by increasing GI transit rate. These results suggest that the bulking effect of fibers is not the sole determining factor mediating defecation function.

The gut microbiota structure changes under constipated conditions. Parthasarathy et al. observed significant increases in bacteria from the Bacteroidota phylum in the colonic mucosa of patients with constipation [32]. These bacteria, which attach to the colonic mucosa, may suppress intestinal motility either through their metabolites or directly [33]. In this study, a moderate increase in the Bacteroidota phylum in the MC group likely contributed to the development of constipation. Furthermore, the MC group exhibited significant decreases in the abundance of six bacterial genera, including *Ruminococcus*, *Oscillibacter*, *Oscillospiraceae_unclassified*, *Alistipes*, *Mucispirillum*, and *Intestinimonas*. *Ruminococcus* is crucial symbionts in the gut ecosystem, playing a vital role in breaking down complex carbohydrates, particularly cellulose and non-cellulosic polysaccharides, and converting them into various nutrients for their hosts [34]. Ren et al. observed a significant reduction in *Ruminococcus* in the fecal samples of constipated children [35]. *Oscillibacter* and *Oscillospiraceae_unclassified*, belonging to the *Oscillospiraceae* family, ferment simple dietary carbohydrates or host glycans, producing beneficial substances like butyrate and leptin [36]. Due to their butyrate-producing capabilities, *Oscillospira* is considered promising candidates for next-generation probiotics [37]. *Mucispirillum* is highly prevalent in the murine gut and tend to colonize the outer mucus layer, enhancing the integrity of the intestinal mucosal barrier [38]. *Intestinimonas* is also known for its butyrate-producing abilities, utilizing lysine and its analogs as substrates [39,40]. He et al. confirmed that lower richness of *Intestinimonas* species increases the risk of constipation in Europeans [18]. *Alistipes* encompasses bacteria with diverse immunological and biochemical pathways associated with several diseases. Previous literature has highlighted its potential in promoting healthy phenotypes and offering protective roles against conditions such as colitis and autism spectrum disorder [41]. Based on the changes in bacterial abundance and their functional characteristics, it can be inferred that the gut microbiota of the constipated mice in this study displayed significant deficiencies in dietary fiber metabolism and butyrate production. PICRUSt analysis revealed a notable decrease in the proportion of carbon and energy metabolism pathways in the MC group, supporting this inference. Results from Mendelian randomization analysis of constipated Europeans’ gut microbiota confirmed that changes in the gut microbiota community contribute to the onset and progression of constipation. And these changes were linked to a reduction in fiber degradation capacity, decreased butyrate production, and compromised mucosal integrity [18,42].

Dietary fiber intervention plays a crucial role in shaping gut microbiota composition and function. In this study, the SDF-S promoted the growth of both *Ruminococcaceae_unclassified* and *Lachnospiraceae_unclassified* (Figure 4A,J), while OB only enhanced the richness of *Lachnospiraceae_unclassified*. These differences suggest that *Ruminococcaceae_unclassified* may have a relatively weaker ability to degrade the β-glycosidic bonds in OB because both SDF-S and OB are primarily composed of glucose but different in their glycosidic bond structure. Additionally, these two bacterial genera are important for carbohydrate degradation in the gut and for butyrate production [43]. Their selective proliferation by these two dietary fibers may also indicate that β-glycosidic bond-containing polysaccharides are more favorable for butyrate production. Furthermore, the SDF-S, AG, and IN demonstrated varying effects on the proliferation of butyrate-producing bacteria such as *Oscillibacter*, *Oscillospiraceae_unclassified*, and *Intestinimonas* (Figure 4D,G,L), providing further evidence for the selective impact of dietary fiber structures on gut microbiota. The reduced abundance of *Mucispirillum* in the constipated mice was reversed across all five dietary fiber groups (Figure 4K), which may indicate a notable improvement in mucosal health [38]. The selective proliferation of gut bacteria by dietary fibers is related to specific carbohydrate-binding molecules encoded by the bacteria and the many carbohydrate-active enzymes that may recognize specific glycosidic bonds [44].

SCFA is the primary metabolite derived from dietary fermentation in the gut, with acetic acid, propionic acid, and butyric acid accounting for over 90% of the total SCFAs produced [45]. These acids can regulate fecal water content by acting as anionic osmotic regulators in the intestine [46]. They are absorbed by colonocytes either through diffusion or active transporters and can activate G-protein coupled receptors GPR41 and GPR4343, exerting multiple physiological effects [47]. These effects include anti-inflammatory actions, promoting colonocyte proliferation, and conditioning intestinal wall peristalsis by regulating the release of gastrointestinal hormones [48,49]. A previous study has shown that acetic and butyric acids, but not propionic acid, exhibit anti-constipation effects, and the SCFA ratio is a better indicator of the host’s constipation state than the SCFA content [27]. Acetic acid is known to increase fecal water content and promote GI transit rate [27]. Butyric acid serves as a fuel for gut epithelial cells and enhances gastrointestinal health by promoting GI motility, reducing inflammation, maintaining the gut barrier, and supporting a healthy microbiome [49,50]. In this study, the positive correlation between the fecal butyric acid ratio and both GI transit rate and 6 h fecal output, as well as the positive correlation between the acetic acid ratio and fecal moisture (Figure 6), further validate these findings. Additionally, the negative correlation between stool moisture and fecal propionic and butyric acid ratios might be attributed to the lower water solubility of these two SCFAs, as well as their inverse quantitative relationship with acetic acid. The negative correlation between the acetic acid ratio and 6 h fecal weight may be related to acetic acid absorption in the large intestine, as previous reports indicate that acetic acid can be absorbed by passive diffusion in the colon and rectum, with absorption inversely related to fecal acetic acid content [51]. These results also highlight the complex impact of fecal SCFAs on defecation function through different mechanisms. The intricate balance and interactions among these SCFAs suggest that their roles in gut health and motility are multifaceted, influencing various physiological processes that contribute to bowel function.

The composition of SCFA is influenced by various aspects of dietary fiber structure, with monosaccharide composition being a primary factor, along with other elements such as glycosidic bond types, molecular weight, and more [52]. The differences in SCFA composition are also a result of dietary fibers’ selective proliferation of bacteria, with the combined effects of SCFAs and microbiota leading to differences in the constipation-relieving efficacy of dietary fibers with different structures. Zhao et al. suggested that arabinose and galactose have stronger abilities to generate butyrate [53], which aligns with the positive correlation between the butyrate ratio and the concentration of arabinose and galactose in this study, as well as the higher butyric acid production observed in the AG group (Figure 1 and Figure 6). Both galactose and arabinose were significantly positively correlated with *Lachnospiraceae_UCG-006*, a core gut bacterium associated with polysaccharides metabolism and SCFA-like butyrate fermentation, which might contribute to the butyric acid production of the AG. Additionally, galactose was significantly positively correlated with *Bacteroides* and *Odoribacter*, both of which were also significantly positively correlated with 6 h fecal weight in the present study (Figure 6). *Bacteroides* is a major carbohydrate-degrading bacterium and a key species rich in carbohydrate-active enzymes, involved in the breakdown of various glycosides [54]. Zhou et al. reported that an increased abundance of this bacterium is associated with a lower risk of constipation [42]. *Odoribacter*, a butyric acid and isoallolithocholic acid producer, has been shown to exert potent antimicrobial effects against Gram-positive bacteria and confers a protective role against the harmful bacteria in the colon [55]. These findings suggest that the selective proliferation of these three genera of bacteria contributes to the AG’s mitigating effect on constipation.

The positive correlation between the fecal acetic acid ratio and glucose concentration, along with the significantly higher acetic acid ratio in the three fibers (SDF-S, OB, and PD) mainly composed of glucose, suggests the preference for acetate fermentation from glucose in dietary fiber. Zhang et al. found that more acetic acid was produced during the fermentation of *L. reuteri*, *L. acidophilus*, and *L. rhamnosus* when glucose was used as the carbohydrate source instead of IN and SDF-S [26], which provides evidence supporting this inference. Glucose is the easiest fermentable monosaccharide in dietary fibers, with a fermentation rate greater than 80% in various fibers [52,54]. This indicates that it can be utilized by most bacteria, and its selective proliferation effect on the gut microbiota is weaker [56]. In this study, no correlation with carbohydrate-degrading bacteria was observed, either. As previously mentioned, dietary fibers primarily polymerized by β-glycosidic bonds may be degraded by *Lachnospiraceae_unclassified*, which could be responsible for producing more butyric acid than fibers with α-glycosidic bonds. Fructose, the main monosaccharide component of IN, was positively correlated with propionic acid in this study, suggesting it promotes propionic acid metabolism, which is consistent with the results of Hernot et al. [57]. Fructose also exhibited a significant positive correlation with the abundance of *Muribaculaceae_unclassified*, a genus that was positively correlated with fecal propionic acid and butyric acid ratios, as well as 6 h fecal weight (Figure 6). Additionally, fructose is positively correlated with the abundance of *Ruminococcus*, indicating that IN intervention reversed the decrease in the abundance of this genus in constipated mice. These results suggest that *Muribaculaceae_unclassified and Ruminococcus* proliferation may be involved in alleviating constipation induced by the IN.

The dietary fibers used in this study also contain various minor monosaccharide components, such as guluronic acid, ribose, and rhamnose, as listed in Table 1. These monosaccharides are present in the side chains of dietary fibers or embedded within the main polysaccharide chains [22,25]. Gut microbiota initially degrades the side chains of polysaccharides before proceeding to the main chains [25,53]. In this study, several beneficial gut bacteria, including *Oscillospiraceae_unclassified*, *Anaerotignum*, and *Lachnospiraceae_unclassified*, were positively correlated with these ingredients, suggesting that these minor components may primarily influence the direction in which dietary fibers regulate gut microbiota composition due to their priority in gut fermentation. Furthermore, Zhao et al. demonstrated that polysaccharides containing side-chains exhibited lower degradation efficiency in the fermentation of gut microbiota than those with linear structures [53]. Our study found that the total SCFA production derived from AG and PD, which are side-chain polysaccharides, was significantly lower than those of the other three dietary fibers. Using total SCFA production as a measure of fermentation degree [58], these results are in line with Zhao et al.’s report [53]. It also suggests that these minor components in dietary fibers may affect the efficacy of dietary fiber in alleviating constipation by influencing the rate and extent of fermentation. However, the specific mechanisms underlying these effects require further investigation.

## 4. Materials and Methods

### 4.1. Materials

AG, IN, and PD with purities of over 90% were purchased from Yuanye Bio-Technology Co., Ltd. (Shanghai, China). OB (≥90% purity) was purchased from Senyo Biotech Co., Ltd. (Huzhou, China). SDF-S was prepared according to our previous study, and its structural information was contained in our published article [26].

SCFA standards (acetic, propionic, and n-butyric acids) were purchased from Sigma-Aldrich Co. LLC (Shanghai, China). Loperamide hydrochloride was obtained from Janssen Pharmaceutical, Ltd. (Xi’an, China). Phenolphthalein was purchased from Aladdin Biochemical Technology Co., Ltd. (Shanghai, China). Gum arabic was purchased from Yonghua Chemical Co., Ltd. (Suzhou, China). Activated carbon was purchased from Macklin Inc. (Shanghai, China). All the other chemicals and reagents used in this study were of analytical grade, purchasing from China National Pharmaceutical Group Co. Ltd. (Shanghai, China).

### 4.2. Determination of the Structural Properties of Fibers

All these fibers underwent the following analysis to confirm their monosaccharide profile, molecular weights, and infrared spectrum information.

The monosaccharaide profile of each fiber was determined using the protocols as follows. The fiber sample was hydrolyzed by 2 mol/L trifluoroacetic acid at 110 °C for 5 h in a sealed tube with nitrogen (N_2_) filling. Then, the reaction mixture was cooled and dried by N_2_ blowing. After adding 0.05 mL of 0.3 mol/L NaOH and 0.05 mL of 0.5 mol/L methanolic 1-pheny-3-methyl-5-pyrazolone solution, the mixture was further incubated at 70 °C for 60 min with N_2_ filling. Standard substances, including rhamnose, arabinose, mannose, ribose, glucose, galactose, fucose, glucuronic acid, and galacturonic acid, were treated identically to the fiber sample. After the incubation, the mixture was neutralized with 0.3 mol/L HCl, then extracted by adding 1.5 mL trichloromethane, mixing thoroughly, and then standing by for 20 min. The supernatants of the triple extraction were collected carefully, combined, and then filtered with 0.45 μm membrane. Ten microliter of the filtered sample was injected into an Agilent C18 column (4.6 mm × 250 mm × 5 μm; Agilent Technologies, Inc., Santa Clara, CA, USA) connected to an HPLC system (1200 Series; Agilent Technologies, Inc., Santa Clara, CA, USA). A chromatographic program was performed as follows: mixed acetonitrile and 0.1 mol/L phosphate buffer (pH 6.8) with the ratio of 18:82 as the mobile phase, column temperature kept at 25 °C, and UV detection set at 254 nm.

The molecule weights of these fibers were determined via size exclusion chromatography coupled with multi-angle laser light scattering (SEC-MALLS, λ = 658 nm, Wyatt Technology Corporation, Santa Barbara, CA, USA). The dietary fiber samples were dissolved in ultra-pure water, and then filtered through a 0.22 μm membrane. An aliquot of 100 μL filtered sample was injected into a TSKgel G5000PWXL column (30 cm × 7.8 mm ID, 10 µm; Tosoh Corporation, Tokyo, Japan) connected with a TSKgel G3000PWXL column (30 cm × 7.8 mm ID, 6 µm; Tosoh Corporation, Tokyo, Japan). The chromatographic conditions were as follows: column temperature of 25 °C; 0.1 mol/L NaNO_3_ as the mobile phase, 0.5 mL/min flow rate. A refractive index detector is employed, with the refractive index increment (dn/dc) value of 0.138 mL/g. The data were analyzed using Astra 7.3.2 software package.

An ATR-IR spectrophotometer (VERTEX70; Shimadzu Corporation, Kyoto, Japan) was used to confirm the chemical bonds and functional groups of the fiber samples by scanning in a range of 4000 cm^−1^ – 600 cm^−1^.

### 4.3. Animals and Experimental Design

A total of 96 male ICR mice (4 weeks, 20 ± 2 g) were purchased from Qizhen Experimental Animals Technology Co., Ltd. (Hangzhou, China; animal license number: SCXK (ZHE) 2022–0022). All experimental procedures were conducted according to the National Institutes of Health Guide for the Care and Use of Laboratory Animals and were approved by the Laboratory Animals Welfare and Ethics Committee of Zhejiang Academy of Agricultural Sciences (Approval number: 2023ZAASLA48). The mice were acclimatized for 7 d and then randomly divided into 8 groups (12 mice per group) as follows: normal control group (NC), model control group (MC), positive treatment group (PT), and five soluble dietary fiber groups (SDF-S, OB, PD, AG, and IN). To induce constipation, mice in the MC and five fiber groups were administered loperamide hydrochloride at a dose of 10 mg/kg body weight (bw) daily, while mice in the NC group were administered saline. Different treatments among groups were conducted during the same period of constipation induction as follows: mice in the NC and MC groups were administered saline, and mice in the PT group were administered phenolphthalein for constipation treatment according to the reported dose of 70 mg/kg bw/d [11]. Mice in the SDF-S, OB, PD, AG, and IN groups were administered the corresponding soluble fibers at doses of 400 mg/kg bw/d according to the results of our previous study [21]. All treatments were conducted by oral gavage with equal volumes for consecutive 14 d, and constipation-inducing preceding drug or soluble –fibers were administered daily. During the experiments, all mice were housed in individually ventilated cages (6 mice per cage, 22 ± 2 °C, relative humidity of 50 ± 5%) under 12/12 h of light/dark cycle, with free access to food and water. Body weights and food consumption were recorded weekly.

### 4.4. Assessing Effects of Polysaccharides on Constipation

#### 4.4.1. SCFA in Feces

After 14 d treatment, the mice were moved into clean and empty cages for 2 h with feed and water available ad libitum, 2 mice from the same group in one cage. The fecal pellets from each cage were collected, sealed, and kept in dry ice immediately and then stored at −80 °C for subsequent analysis.

SCFA was determined according to the method of Zhao et al. [59]. Briefly, 0.2 g of fecal sample was mixed with 2 mL of cooled, sterile deionized water, vortexed for 5 min, and then centrifuged at 10,000 rpm at 4 °C for 20 min (Cence H2100R; XiangYi Centrifuge Instrument Co., Ltd., Changsha, China). After that, an aliquot of 0.5 mL of supernatant and 0.1 mL of crotonic acid-metaphosphoric acid solution were combined for acidification at −20 °C for 24 h. The acidified supernatant was thawed and centrifuged at 12,000 rpm at 4 °C for 8 min. The consequent supernatant was filtered through a 0.22 μm membrane and used for SCFA content detection.

The detection was conducted using gas chromatography (GC-2010 plus; Shimadzu Corporation, Kyoto, Japan) along with a DB-FFAP column (30 m × 0.53 mm × 5 μm; Agilent Technologies, Inc., Santa Clara, CA, USA) and a hydrogen (H_2_) flame ionization detector. The injection volume was 1.0 μL and N_2_ was used as the gas carrier at a flow rate of 12.0 mL/min. The flow rates of air, H_2_, and N_2_ in the detector were 400.0, 40.0, and 30.0 mL/min, respectively. The temperature of the injector and detector was kept at 250 °C. The oven temperature program was as follows: initial column temperature of 70 °C, increased to 180 °C at 15 °C/min and increased to 240 °C at 40 °C/min.

#### 4.4.2. Defecation Parameters

On the fifteenth day of the experimental treatment, the mice in each group were given a meal of 5% activated carbon suspension by gavage after 16 h fasting but free access to drinking water. For assaying the defecation function, mice from each group were divided into two parts randomly and participated in the following two trials.

##### Weight and Moisture of Stools

After the activated carbon meal, six mice were randomly picked out from each group and were kept in the clean and empty cages (2 mice in one cage) for a 6 h period, with free access to feed and drinking water ad libitum. During this period, all fecal pellets were collected and weighed, and the moisture content was determined based on the difference between wet and dry weights/the wet weights ×100.

##### Small Intestinal Propulsion Trial

Thirty minutes after the activated carbon meal, the rest 6 mice in each group were sacrificed by cervical dislocation. Blood samples were collected from the orbits, and the separated serum was used for GI hormone assays. The entire intestine, from the pylorus to the cecum, was excised quickly and gently spread on a piece of filter paper. The total length of the intestine (D1) and the distance between the pylorus and the front of the black digesta (D2) were measured. The GI transit rates were calculated as follows: D2/D1 × 100. The cecum contents were collected, sealed, and placed on dry ice immediately for gut microbiota analysis.

#### 4.4.3. Serum Gastrointestinal Regulatory Hormones

The blood samples were stored at 4 °C for 2 h, then centrifuged at 2500 rpm, 4 °C for 10 min for serum separation. GI regulatory hormones, including motilin (MTL), gastrin (GAS), substance P (SP), somatostatin (SS), and vasoactive intestinal peptide (VIP), were determined using the corresponding enzyme-linked immunosorbent assay (ELISA) kits (Lengdon Biotech. Co., Ltd., Shanghai, China).

#### 4.4.4. 16S rDNA Gene Sequencing of Caecum Contents

The caecum contents of each mouse were transported to the laboratory of LC-Bio Technology Co., Ltd., Hangzhou, Zhejiang Province, China, for 16S rDNA gene sequencing. All experiments, including DNA extraction, quantitation, amplification, purification, etc., were performed according to the standard protocols and methods of the LC-Bio. Polymerase chain reaction (PCR) amplification of the V3 and V4 regions of bacterial 16S rDNA genes was carried out using the primers 341F (5′-CCTACGGGNGGCWGCAG-3′) and 805R (5′-GACTACHVGGGTATCTAATCC-3′) and were sequenced on a NovaSeq PE250 platform (Illumina Inc., San Diego, CA, USA). The raw data collected from sequencing were further processed (sequence merging, quality filtering, chimeric sequence filtering, and dereplication) to obtain the ASV table and sequence using various software, including FLASH (v 1.2.7, https://ccb.jhu.edu/software/FLASH/index.shtml, accessed on 29 November 2022), fqtrim (v 0.94, http://ccb.jhu.edu/software/fqtrim/, accessed on 29 November 2022), Vsearch software (v 2.3.4, https://github.com/torognes/vsearch, accessed on 29 November 2022), and QIIME2 (v 2019.7, https://qiime2.org/, accessed on 29 November 2022). Bioinformatics analysis including community composition, diversity, and correlations with other constipation-related biological parameters was performed using QIIME2, BLAST (v 2.15.0, https://blast.ncbi.nlm.nih.gov/Blast.cgi, accessed on 29 November 2022), and the SILVA database (release 138, https://www.arb-silva.de/documentation/release-138, accessed on 29 November 2022). Function prediction of various gut microbiota was conducted by using PICRUSt2 (v2.5.2, https://github.com/picrust/picrust2/, accessed on 5 December 2024) based on the representative sequences of amplicon sequence variants (ASV). The statistics analysis (Kruskal–Wallis test) and graphing were implemented by using R package (v 3.5.2).

### 4.5. Statistical Analysis

Data obtained from all the measurements were expressed as mean ± standard deviation. Statistical analyses were performed using SPSS 26.0 (IBM SPSS Inc., Chicago, IL, USA). Differences between groups were determined using the method of completely randomized one-way analysis of variance, followed by Duncan’s multiple comparison test, or with Welch’s test followed by Games-Howell multiple comparison for heteroscedastic variables. A probability of *p* < 0.05 was regarded as statistically significant. The figures, other than those related to bioinformatics analysis, were created using OriginPro 2022 software (OriginLab, Co., Northampton, MA, USA).

## 5. Conclusions

In this study, the anti-constipation effects of five dietary fibers with varying monosaccharide compositions and glycosidic bonds were compared. The mechanisms were further explored by examining the relationships between defecation function, dietary fiber’s structural characteristics, gut microbiota composition, and SCFA profiles. The results indicated that four dietary fibers promoted GI transit in the following order: AG > OB = SDF-S > IN. AG and IN showed higher 6 h stool weights compared to the other three dietary fibers, while PD only showed a tendency to increase stool moisture. Therefore, AG could be considered a more effective laxative agent for alleviating constipation than others. This work also revealed how differences in monosaccharides and glycosidic bonds affect the gut microbiota and SCFA composition in constipated mice. It highlighted the complex interactions between changes in these bacteria and SCFAs and their impact on defecation function parameters. The study clarified the important role of galactose, arabinose, and β-glycosidic bonds in promoting butyrate production and associated bacterial genera, which play a crucial role in improving constipation. It also suggested that certain monosaccharides in side chains influence the degree of polysaccharide fermentation and the direction of microbiota regulation, thereby affecting the efficacy of dietary fibers in relieving constipation. These findings provide new insights into the differential effects of dietary fibers on constipation relief, aiding in the development of more effective fiber-based treatments.

## Figures and Tables

**Table 1:** Monosaccharide composition and average molecular weight of five soluble dietary fibers: soluble dietary fiber from steamed sweet potato (SDF-S), Oat beta-glucan (OB), polydextrose (PD), inulin (IN), arabinogalactan (AG).

Items	SDF-S	OB	PD	IN	AG
Molecular weight (kDa)	5.30	101.24	3.01	3.35	48.07
Monosaccharide composition (mg/g)					
Guluronic acid	23.94	0.62	1.20	0.47	0.34
Mannose	4.00	6.05	0.57	0.73	2.41
Ribose	0.87	0.15	0.30	0.05	0.18
Rhamnose	5.65	0.53	0.71	N.D.	0.74
Glucuronic acid	1.44	1.98	0.06	N.D.	5.03
Galacturonic acid	25.48	1.22	1.43	1.24	0.06
Glucose	846.85	826.24	909.06	278.73	2.16
Galactose	30.03	11.68	1.37	6.09	864.58
Xylose	1.75	40.04	0.96	N.D.	N.D.
Arabinose	12.39	48.59	0.65	0.91	99.20
Fucose	1.13	1.17	N.D.	0.92	1.42
Fructose *	/	/	/	675.83	/

* Fructose content determination was only conducted in the inulin sample. N.D. = not detected.

**Table 2:** Defecation function of constipated mice after 14 d intervention with different fibers.

Groups	6 h Stool Weight (g)	Stool Moisture (%)	GI Transit Rate (%)
NC	0.60 ± 0.31 ^bc^	68.86 ± 2.51 ^a^	88.35 ± 5.17 ^a^
MC	0.40 ± 0.09 ^c^	30.42 ± 3.98 ^d^	33.32 ± 7.32 ^d^
PT	0.83 ± 0.12 ^ab^	45.56 ± 5.37 ^bc^	63.23 ± 7.67 ^b^
SDF-S	0.83 ± 0.12 ^ab^	49.53 ± 2.31 ^bc^	58.92 ± 7.78 ^b^
OB	0.77 ± 0.29 ^ab^	46.50 ± 4.50 ^bc^	59.67 ± 10.62 ^b^
PD	0.76 ± 0.08 ^ab^	50.35 ± 3.64 ^b^	37.82 ± 3.95 ^d^
AG	1.12 ± 0.21 ^a^	46.15 ± 3.36 ^bc^	68.10 ±4.88 ^b^
IN	0.99 ± 0.20 ^a^	42.68 ± 2.04 ^c^	49.36 ± 7.84 ^c^

Note: Different lowercase letters in the same column indicate significant differences at *p* < 0.05. NC = normal control group; MC = model control group; PT, SDF-S, OB, PD, AG, and IN signify the groups treated with phenolphthalein, soluble dietary fiber from steamed sweet potato (SDF-S), Oat beta-glucan (OB), polydextrose (PD), inulin (IN), and arabinogalactan (AG), respectively.

**Table 3:** Serum levels of motilin (MTL), gastrin (GAS), substance P (SP), vasoactive intestinal peptide (VIP), and somatostatin (SS).

Groups	MLT (ng/L)	GAS (ng/L)	SP (ng/mL)	VIP (ng/L)	SS (ng/L)
NC	170.07 ± 38.22	254.21 ± 24.48 ^bc^	1.24 ± 0.17 ^a^	337.00 ± 55.65 ^d^	174.34 ± 13.32 ^c^
MC	163.09 ± 26.92	205.23 ± 54.06 ^c^	0.43 ± 0.09 ^d^	613.64 ± 57.77 ^a^	255.73 ± 15.85 ^a^
PT	182.51 ± 29.77	351.85 ± 33.14 ^a^	1.29 ± 0.05 ^a^	482.35 ± 53.87 ^bc^	168.34 ± 12.21 ^c^
SDF-S	138.27 ± 30.12	356.13 ± 52.96 ^a^	0.87 ± 0.16 ^b^	482.09 ± 83.19 ^bc^	172.51 ± 19.32 ^c^
OB	146.04 ± 33.76	293.47 ± 48.30 ^ab^	0.75 ± 0.14 ^bc^	330.75 ± 43.21 ^d^	172.12 ± 9.26 ^c^
PD	158.82 ± 13.85	263.93 ± 20.68 ^bc^	0.77 ± 0.02 ^bc^	380.90 ± 74.65 ^cd^	193.99 ± 15.77 ^bc^
AG	176.77 ± 50.81	262.16 ± 43.13 ^bc^	0.66 ± 0.16 ^bc^	527.47 ± 47.90 ^ab^	184.34 ± 18.84 ^bc^
IN	201.40 ± 31.86	279.57 ± 24.70 ^b^	0.57 ± 0.19 ^cd^	477.51 ± 61.48 ^bc^	201.19 ± 20.40 ^b^

Note: Different lowercase letters in the same column indicate significant differences at *p* < 0.05.

## Data Availability

The data presented in this study are available upon request from the corresponding author. The data are not publicly available due to privacy restrictions.

## Supplementary Materials

The following supporting information can be downloaded at: https://www.mdpi.com/article/10.3390/ijms26031236/s1.
