# Supplementary material for: Different Efficacy of Five Soluble Dietary Fibers on Alleviating Loperamide-Induced Constipation in Mice: Influences of Different Structural Features
Source: Int J Mol Sci. 2025 Jan 31;26(3):1236. doi: 10.3390/ijms26031236 (PMC11818199; doi:10.3390/ijms26031236)
Supplement: Supplementary file 1 [file ijms-26-01236-s001.zip › ijms-3336219-supplementary.pdf]

# Supplementary materials

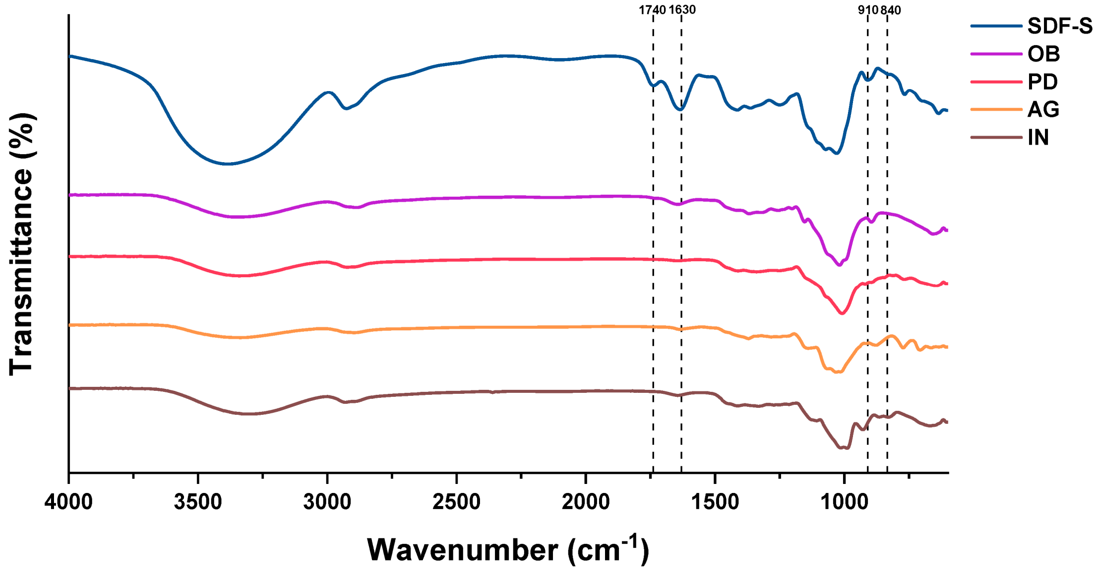


**Figure S1. Infrared spectra of soluble dietary fiber from steamed sweet potato (SDF-S), oat β-glucan (OB), polydextrose (PD), arabinogalactan (AG), and inulin (IN).**
